# Surface Modification and Sugar Infusion of Freeze-Dried Hawthorn via Combined Vacuum-Ultrasound Treatment

**DOI:** 10.3390/foods15162873

**Published:** 2026-08-17

**Authors:** Fei Shi, Jiguang Liu, Anlun Sun, Yuchuan Wang, Bo Wang

**Affiliations:** 1 School of Food Science and Engineering, Jiangsu University, Zhenjiang 212013, China; sf152651@126.com; 2 Shandong Commune Union Food Co., Ltd., Linyi 276034, China; ljg0321@163.com (J.L.); sunanlun007@126.com (A.S.); 3 School of Food Science and Technology, Jiangnan University, Wuxi 214122, China; wyc453@163.com

**Keywords:** hawthorn, vacuum impregnation, ultrasonic impregnation, vacuum drying, microstructure, uniformity

## Abstract

Hawthorn (*Crataegus pinnatifida* Bunge) is a medicinal and edible fruit whose sour taste and dense surface structure limit its application in food industries. Conventional sugar impregnation has long processing times and uneven sugar distribution. However, the combined effects of vacuum impregnation (VI) and ultrasonic impregnation (UI) on infusion uniformity in freeze-dried hawthorn remain unclear. This study therefore aimed to elucidate the mechanisms by which combined vacuum and ultrasonic impregnation (VI-UI) affect surface structure and sugar infusion uniformity, and to test the hypothesis that VI-UI can achieve uniform sugar distribution while maintaining an intermediate sugar content. Results showed that the VI-UI treatment (195 min) disrupted cell structures and expanded intercellular spaces in hawthorn, thereby achieving a uniform sugar infusion at an intermediate sugar content of 47%. The ultrasound treatment increased surface porosity from 16.8% to 19.3%. The VI-UI treatment achieved the highest overall uniformity index (OUI = 79.65), higher than vacuum impregnation (OUI = 50.99) and ultrasound impregnation (OUI = 60.69). In conclusion, VI-UI offers a promising strategy for producing uniformly infused, high-quality preserved hawthorn products by combining the efficiency of VI with the uniformity of UI.

## 1. Introduction

Hawthorn (*Crataegus pinnatifida* Bunge) is a medicinal and edible fruit that is widely distributed throughout the Northern Hemisphere and contains a wide range of bioactive compounds, such as flavonoids, polyphenols and organic acids. It exhibits multiple health-promoting effects, such as digestion enhancement, anti-inflammatory and antioxidant activities, and cardiovascular protection [1]. Despite its high nutritional value and distinctive flavor, fresh hawthorn has high acidity and poor postharvest storability, necessitating further processing to enhance its added value.

Hawthorn fruit has a dense surface structure, a thick waxy layer, deep coloration, and compact internal cells [2,3]. However, fresh hawthorn is seasonally available and has a short postharvest storage life, making it difficult to maintain consistent raw material quality. Freeze-drying overcomes this limitation by enabling the preparation and storage of uniform material, ensuring experimental reproducibility. In addition, vacuum impregnation is inherently most effective for porous materials, and the dense structure of fresh hawthorn substantially limits mass transfer efficiency. Therefore, freeze-dried hawthorn was used in this study to improve mass transfer and tissue uniformity prior to impregnation. This porous structure in the hawthorn tissue, formed by sublimation of the ice crystals during freeze-drying, allows the subsequent permeation of sugar [4]. However, there are several disadvantages of conventional impregnation such as lengthy sugar infusion time, non-uniform penetration of the sugar solution and high sugar content in the end product. Such problems can result in unwanted loss of native flavor and nutrition, negatively impacting the quality of the products [5,6].

Atmospheric pressure impregnation (API), microwave impregnation (MI), vacuum impregnation (VI) and ultrasonic impregnation (UI) are the common sugar impregnation techniques. This study focused on VI and UI, as these two methods offer distinct and potentially complementary mass transfer mechanisms. Vacuum impregnation (VI) removes gas from the porous structure of food material under vacuum conditions. Upon pressure restoration, the sugar solution penetrates the pores via capillary action, reducing mass transfer resistance and increasing the impregnation rate [7,8,9]. Ultrasonic impregnation (UI) utilizes the physical effects of ultrasound, which disrupts the cell structure by creating micro-channels via cavitation, thereby promoting solution diffusion and improving mass transfer [10,11,12]. To the best of our knowledge, existing studies on VI and UI have mostly been performed separately, with each technique exhibiting unique merits: VI provides high sugar-loading efficiency, whereas UI improves sugar distribution uniformity [7,8,9,10,11,12]. Nevertheless, both approaches possess inherent limitations. While VI facilitates high sugar loading, it inevitably leads to an uneven impregnation pattern, characterized by a distinct concentration gradient from the interior to the exterior—a feature that compromises product uniformity. In contrast, UI achieves homogeneous sugar distribution but suffers from low total sugar uptake, limiting final product quality. These drawbacks cannot be mitigated by merely tuning individual processing parameters, since VI and UI follow fundamentally different mass-transfer mechanisms: pressure-driven capillary action for VI and cavitation-induced surface disruption for UI. Combining VI and UI holds great promise to circumvent these respective shortcomings by taking advantage of their complementary mechanisms. VI effectively delivers sugar into the porous matrix, while UI subsequently redistributes sugar evenly across the plant tissue. Notably, the porous architecture formed by freeze-drying alone cannot guarantee uniform sugar impregnation; it only provides a basic structural framework. Despite such promising potential, the combined VI-UI treatment—especially its influence on infusion uniformity within freeze-dried hawthorn—has not been systematically explored, and its underlying mechanisms remain poorly understood. This knowledge gap hinders the development of more efficient and homogeneous impregnation processes for hawthorn-derived products.

Therefore, this study aimed to investigate the effects of combined VI-UI treatment on sugar infusion uniformity in freeze-dried hawthorn and to elucidate the underlying microstructural mechanisms. The central hypothesis was that VI-UI would achieve more uniform sugar distribution than VI or UI alone by combining the high sugar loading efficiency of VI with the uniformity-enhancing effect of UI, while maintaining an intermediate sugar content.

The findings of this study provide a mechanistic basis for the combined vacuum-ultrasound impregnation process, which is relevant to preserved hawthorn production. Freeze-drying was employed as a pretreatment to improve mass transfer and tissue uniformity, allowing systematic evaluation of the impregnation mechanisms. The complementary roles of VI and UI—efficient sugar loading and uniform distribution, respectively—are transferable principles that can guide process adjustment for fresh fruit impregnation. For industrial application, these findings offer practical value in two key aspects. First, the mechanistic understanding of VI and UI enables targeted adjustment of processing parameters (e.g., pressure, ultrasound power, and treatment time) to suit different raw material characteristics, reducing the need for extensive trial-and-error in process development. Second, the demonstrated improvement in sugar distribution uniformity directly addresses a common quality issue in preserved fruit production, offering a feasible pathway to enhance product consistency and market value. While direct application to fresh hawthorn would require further validation, the established framework provides actionable insights for industrial process design and improvement.

## 2. Materials and Methods

### 2.1. Materials

Fresh hawthorn (*Crataegus pinnatifida* Bunge, cv. ‘Dajinxing’) was purchased from a local orchard in the Yimeng Mountains, Shandong Province, China. De-cored freeze-dried hawthorn (*Crataegus pinnatifida* Bunge, cv. ‘Dajinxing’) with a moisture content of 12% was obtained from a local industry in Linyi City, Shandong Province, China. Sucrose (purity ≥ 99.5%) was purchased from Nanning Mingyang Sugar Co., Ltd. (Nanning, Guangxi, China). Glucose (purity ≥ 99.5%) was purchased from Yunnan Xingguanyuan Food Co., Ltd. (Kunming, Yunnan, China). High-fructose corn syrup (solid content ≥ 77%) was purchased from Hubei Qianfengxiang Food Co., Ltd. (Wuhan, Hubei, China). Anhydrous copper sulfate was purchased from Shanghai Aladdin Biochemical Technology Co., Ltd. (Shanghai, China). D-(+)-Glucose (anhydrous) and potassium sodium tartrate were purchased from Shanghai YuanYe Biotechnology Co., Ltd. (Shanghai, China). Anhydrous ethanol, hydrochloric acid, and sulfuric acid were purchased from Kunshan Jincheng Reagent Co., Ltd. (Kunshan, Jiangsu, China). Galacturonic acid, methyl red, methylene blue, coumarin, sodium hydroxide, and potassium ferrocyanide were purchased from Shanghai Macklin Biochemical Technology Co., Ltd. (Shanghai, China). Diluted iodine solution was purchased from Taizhou Moer Teaching Instrument Equipment Co., Ltd. (Taizhou, Jiangsu, China).

### 2.2. Sugar Impregnation and Vacuum Drying

De-cored freeze-dried hawthorn was used as the raw material and processed according to the following methods.

VI method: A sugar solution composed of high-fructose corn syrup, sucrose, glucose, and purified water was prepared at a final concentration of 64.58 ± 1.02 °Brix [6]. The sugar concentration was measured using a handheld refractometer (0–90 °Brix, Hunan Yunyi E-commerce Co., Ltd., Changsha, Hunan, China), which was calibrated with deionized water prior to use. Vacuum impregnation was carried out using vacuum sugar impregnation equipment (SHZ-D (III), Hunan Yunyi E-commerce Co., Ltd., Changsha, Hunan, China) under a vacuum pressure of –0.08 MPa at 85 °C for 90 min.

UI method: Hawthorn samples were pre-immersed in a low-concentration sugar solution (A sugar solution consisting of 15% glucose and 85% purified water (*w*/*w*) was prepared, with a measured concentration of 15.75 ± 0.94 °Brix) at 85 °C for 90 min. Subsequently, a high-concentration sugar solution (64.58 ± 1.02 °Brix) was cooled to 20–25 °C. Ultrasonic-assisted sugar impregnation was then performed using an ultrasonic cell disruption instrument (JY92-IIN, Ningbo Xinzhi Biotechnology Co., Ltd., Ningbo, Zhejiang, China) equipped with a φ6 mm titanium alloy probe. The instrument was operated at an ultrasonic power of 325 W (below the maximum output of 650 W) and a fixed frequency of 20–25 kHz for 15 min, with the probe immersed directly into 450 mL of the sugar solution.

VI-UI method: A low-concentration sugar solution (15.75 ± 0.94 °Brix) was prepared. Hawthorn samples were pre-immersed in this solution at 85 °C for 90 min. Thereafter, the samples were subjected to vacuum impregnation in a high-concentration sugar solution (64.58 ± 1.02 °Brix) under a vacuum pressure of –0.08 MPa at 85 °C for 90 min. A fresh low-concentration sugar solution (15.75 ± 0.94 °Brix) was then prepared and cooled to 20–25 °C to serve as the ultrasonic medium. The hawthorn samples were removed from the previous solution, placed in a 500 mL beaker, and immersed in 450 mL of the cooled sugar solution, followed by ultrasonic treatment at 325 W for 15 min.

It should be noted that the 85 °C treatment was applied to reduce syrup viscosity and facilitate mass transfer, and was not performed simultaneously with the ultrasound treatment. The ultrasound treatment was conducted at 20–25 °C.

The sugar-impregnated hawthorn samples were vacuum-dried at 2000 Pa and 60 °C for 7 h.

### 2.3. Three-Dimensional Cell Imaging

An ultrasonic cell disruption instrument (JY92-IIN, Ningbo Xinzhi Biotechnology Co., Ltd., China) was used to treat fresh hawthorn samples (fresh hawthorn samples were used for the initial screening of ultrasonic power and time to enable clearer visualization of cellular responses). The instrument has a maximum output of 650 W, and the power output is adjustable as a percentage of this maximum. To screen for a suitable ultrasonic power, samples were treated at different power levels (0%, 30%, 50%, 70%, and 100% of maximum power, corresponding to 0, 195, 325, 455, and 650 W) for 15 min. Separately, to identify a suitable treatment duration, samples were treated at 325 W (the power selected from the previous screening) for different durations (0, 5, 10, 15, 20, and 25 min).

Slide specimens were prepared according to the method described by Dong et al. and Huang et al. [13,14]. The cover glass and slide were cleaned with gauze, and a drop of deionized water was placed on the cover glass. A small incision was made on the pericarp of the ultrasonicated hawthorn sample. Then the pericarp was carefully peeled off using forceps. An irregular tissue piece (approximately 2.5 mm × 2.5 mm) was taken from the edge. It was placed flat in a water droplet. This was done to avoid overlapping. Forceps were used to hold the cover glass. Afterwards, it was carefully lowered over the water droplet. This was done to avoid the formation of bubbles. Iodine solution was then applied to one side of the cover glass using a dropper, and a piece of blotting paper was placed on the opposite side to draw the solution across the specimen, ensuring complete penetration. A high-resolution 3D cell imaging system (THUNDER Imager 3D, Leica Microsystems, Wetzlar, Germany) was used to image the prepared slides.

Rehydrated freeze-dried hawthorn closely resembles fresh tissue in terms of macroscopic physical properties and internal liquid environment [15,16]. Notably, because softer tissues exhibit a lower cavitation threshold and higher cavitation activity under ultrasound [17], the selected parameters determined using fresh tissue are applicable to rehydrated samples.

### 2.4. Measurement of Surface SEM

For SEM observation: two types of samples were prepared: (1) fresh hawthorn samples subjected to ultrasonic treatment, followed by freeze-drying to remove moisture for SEM analysis, and (2) freeze-dried hawthorn samples (prepared as described in Section 2.2) after sugar impregnation (VI, UI, or VI-UI) and vacuum drying. All samples were cut into small pieces (thickness < 1 cm, diameter < 3 cm) and sputtered with gold [18]. The surface morphology was observed using two different scanning electron microscopes: a Regulus 8100 (Hitachi, Ltd., Tokyo, Japan) and a SEM5000Pro (CIQTEK Co., Ltd., Hefei, Anhui, China).

### 2.5. Determination of Total Sugar Content

Six sugar-impregnated hawthorn samples were randomly selected from each treatment group for total sugar content determination according to the literature [19]. Briefly, each sample (10 g) was crushed and soaked in deionized water for 1.5 h. The mixture was then transferred to a 250 mL volumetric flask, neutralized with NaOH solution using methyl red as an indicator, and diluted to the mark with deionized water. The resulting solution was used for titration. Fehling’s reagent A and B (5 mL each) were placed in a conical flask together with glass beads and brought to a boil. The sample solution was added dropwise at a rate of one drop per 2 s until the blue color disappeared. The volume of sample solution consumed was recorded, and triplicate measurements were performed for each sample. The total sugar content was calculated using Equations (1) and (2).

The glucose equivalent mass of 10 mL of Fehling’s reagent was calculated as:

(1) $$M=\frac{m_{0}\times V_{0}}{250}$$

where M is the glucose equivalent mass of 10 mL Fehling’s reagent (g), $m_{0}$ is the glucose mass (g), and $V_{0}$ is the titration volume (mL).

The total sugar content (X, g/100 g) was then calculated as:

(2) $$X=\frac{M\times V_{1}\times V_{3}}{m\times V\times V_{2}}\times 100$$

where $V_{1}$ is the diluted sample volume (mL), $V_{2}$ is the aliquot volume taken (mL), $V_{3}$ is the volume after hydrolysis (mL), m is the sample mass (g), and V is the titration volume (mL).

### 2.6. Determination of the Cross-Sectional Sugar Concentration Gradient

**Figure 1:**
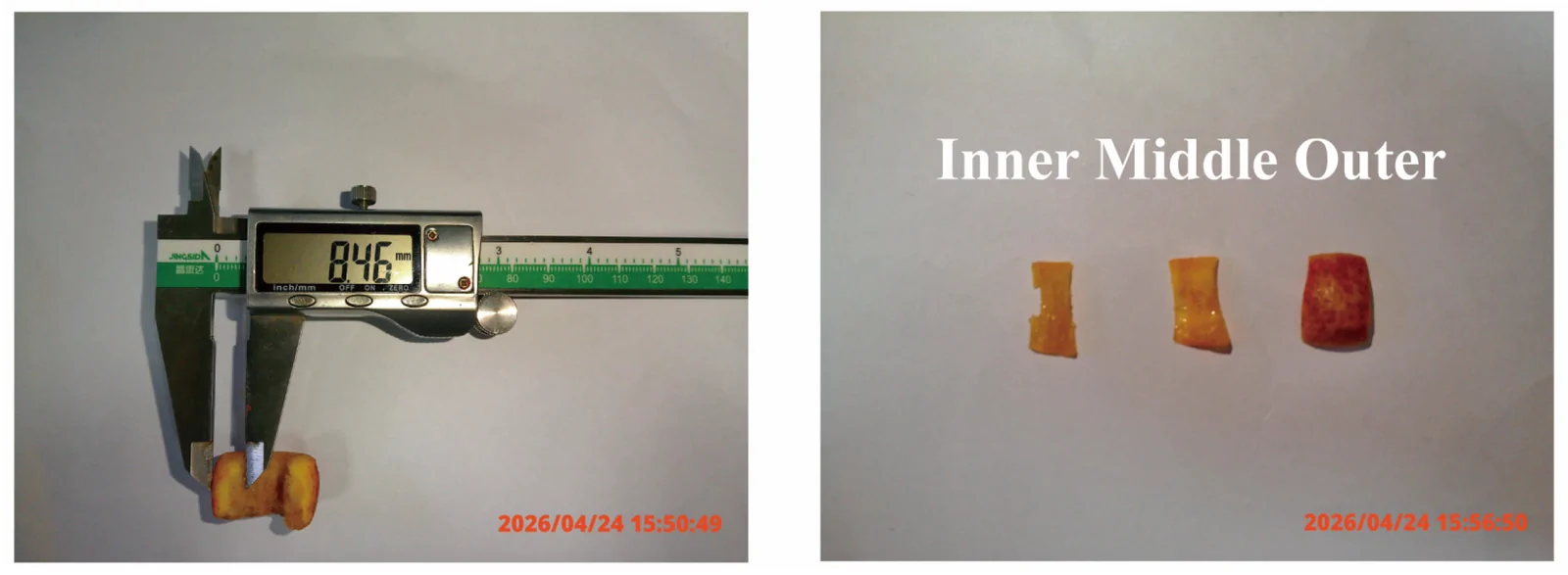
Cross-sectional view of a hawthorn sample showing the inner, middle, and outer regions.

Six sugar-impregnated hawthorn samples were randomly selected from each treatment group. Each sample was placed upright on a plate and sectioned into small pieces. The thickness of each piece was measured using a vernier caliper. Based on the measured thickness, each sample was divided into three concentric regions, for example, the inner region (0–2.8 mm from the center), the middle region (2.8–5.6 mm from the center), and the outer region (5.6–8.4 mm from the center) as illustrated in Figure 1. The total sugar content of each region was determined using the same method described in Section 2.5.

### 2.7. Measurement of Sugar Impregnation Rate and Sample Weight Gain

The sugar impregnation rates obtained from different impregnation methods and the corresponding weight gain of freeze-dried hawthorn samples (before vs. after sugar impregnation) were evaluated according to the methods described by Li et al. [11] and Demir and Alpaslan [20,21], respectively.

Sugar impregnation rate determination: freeze-dried hawthorn samples without any pretreatment were completely submerged in the sugar solution and treated using VI, UI, or VI-UI methods. Samples were removed at scheduled time points (for all treatment steps except the UI step, samples were taken every 10 min; for the UI step, every 3 min), blotted dry with filter paper to remove surface solution, and weighed. The process was continued until no further weight change was observed. Three independent replicates were performed for each treatment group.

Weight gain determination: six freeze-dried hawthorn samples were randomly selected from each treatment group. Each sample was weighed before sugar impregnation ($M_{\mathrm{before}}$) and again after vacuum drying (following the procedure described in Section 2.2) ($M_{\mathrm{after}}$). The weight increase rate was calculated using Equation (3):

(3) $$R=\frac{M_{\mathrm{after}}-M_{\mathrm{before}}}{M_{\mathrm{before}}}\times 100\%$$

where R is the weight increase rate (%), $M_{\mathrm{before}}$ is the weight of freeze-dried hawthorn before soaking (g), and $M_{\mathrm{after}}$ is the weight of hawthorn after drying (g).

### 2.8. Determination of Water Activity and Water Distribution

Water activity: six sugar-impregnated hawthorn samples were randomly selected from each treatment group. Each sample was cut along the central line and divided into three layers (inner, middle, and outer) according to the method described in Section 2.6. Each layer was placed in a separate dish and equilibrated at 25 °C for 30 min before measurement. Water activity was measured using a water activity meter (HD-6, Wuxi Huakuo Instrument and Meter Co., Ltd., Wuxi, Jiangsu, China) calibrated with saturated salt solutions (MgCl_2_ and NaCl). For comparison, water activity was also measured on whole (unsectioned) samples. Three replicate measurements were performed per sample, and results were expressed as mean ± standard deviation.

Water distribution: sugar-impregnated hawthorn samples from different treatment groups were analyzed for water distribution using a low-field nuclear magnetic resonance (NMR) imaging analyzer (NMI20-060VJS-I, Suzhou Numin Analytical Instruments Co., Ltd., Suzhou, Jiangsu, China) with a magnetic field strength of 0.5 T (corresponding to a ^1^H resonance frequency of 20 MHz). Samples were placed in a 20 mm diameter NMR tube and equilibrated to 32 °C for 10 min prior to measurement. The instrument parameters were set as follows: measurement temperature of 32 °C, echo time (TE) of 13.42 ms, repetition time (TR) of 1000 ms, 16 scans per sample, and a 90° pulse width of 13 µs. The images acquired were treated with the software of the instrument for pseudo-color enhancement, according to values of water content from blue (low) to red (high). Three samples per treatment group were analyzed [22].

### 2.9. Assessment of Overall Uniformity

Five quality indicators—total sugar content, hardness, moisture content, pectin content, and color difference (Δ*E*)—were selected to evaluate the overall uniformity of sugar-impregnated hawthorn samples. To eliminate unit effects, the coefficient of variation (*CV*) for each indicator was calculated using Equation (4) [23].

(4) $${CV}_{i}=\frac{\sigma_{i}}{\mu_{i}}\times 100\%$$

where $\sigma_{i}$ represents the standard deviation, and $\mu_{i}$ represents the mean value.

The *CV* values were then converted into uniformity scores on a scale of 0 to 100 according to the following rules [24]:

Score = 100 when CV≤5% (excellent uniformity)

Score = 0 when CV≥20% (unacceptable uniformity)

Score = $100-\frac{\left(CV-5\right)\times 100}{15}$ when 5%<CV<20% (linear interpolation)

An overall uniformity score was calculated using weighted summation [25]. Weights were assigned based on each indicator’s contribution to product quality as follows: total sugar content 30%, hardness 20%, moisture content 25%, pectin content 15%, and color difference 10% (summing to 100%). The overall uniformity index (OUI) was then calculated using Equation (5):

(5) $$\mathrm{OUI}=\sum_{i=1}^{4}\left(S_{i}\times \omega_{i}\right)$$

where $S_{i}$ is the uniformity score of the i-th indicator, and $\omega_{i}$ is the corresponding weight, with $\sum \omega_{i}=1$ (100%). The OUI ranges from 0 to 100, with higher values indicating better overall uniformity. Based on the OUI, the product uniformity was classified into four grades [26]:

Excellent: OUI ≥ 70;

Good: 55 ≤ OUI < 70;

Medium: 40 ≤ OUI < 55;

Poor: OUI < 40.

### 2.10. Determination of Hardness, Moisture Content, Pectin Content, and Color Differences

The hardness of the sugar-impregnated hawthorn samples was measured using a food property tester (TA.XT Plus, Stable Micro Systems Ltd., Godalming, UK). An SMS P/2 probe was used with a contact force of 5 g. The pre-test speed was 3 mm/s, the test speed was 2 mm/s, the post-test speed was 10 mm/s, and the deformation was set to 75% [27].

The moisture content of the sugar-impregnated hawthorn samples was determined using the direct drying method [28]. Six samples were randomly taken from each group and dried in an oven at 105 °C until a constant weight was reached. The moisture content was then calculated using Equation (6).

(6) $$X=\frac{m_{1}-m_{2}}{m_{1}-m_{3}}\times 100\%$$

where X is the water content (%), $m_{1}$ is the weight of the weighing bottle with the hawthorn samples before drying (g), $m_{2}$ is the weight after drying (g), and $m_{3}$ is the weight of the empty weighing bottle (g).

Pectin content was determined by the carbazole-sulfuric acid spectrophotometric method [29] with slight modifications. Briefly, sugar-impregnated hawthorn samples were homogenized, mixed with water, frozen, and then extracted with anhydrous ethanol. The precipitate was washed with 67% ethanol until no Muller-Voss reaction was obtained. The purified precipitate was hydrolyzed with 0.5% sulfuric acid at 85 °C for 60 min, and the resulting solution was reacted with coumarin ethanol solution and sulfuric acid. Absorbance was measured at 525 nm, and pectin content (as galacturonic acid equivalent) was calculated using a standard curve (Supplementary Figure S1). A blank sample was prepared using deionized water.

The color differences (L*, a*, b*) of sugar-impregnated hawthorn samples were measured using a color difference meter (NR20XE, Guangdong Sanen Time Intelligent Technology Co., Ltd., Guangzhou, Guangdong, China) calibrated against a standard white plate. The total color difference (∆*E*) was calculated using Equation (7) [30].

(7) $$\Delta E^{*}=\sqrt{\Delta L^{*2}+\Delta a^{*2}+\Delta b^{*2}}$$

where $\Delta L^{*}$, $\Delta a^{*}$, and $\Delta b^{*}$ are the differences in $L^{*}$, $a^{*}$ and $b^{*}$ values between freeze-dried and hawthorn samples, respectively, and ΔE is the total color difference. A ΔE value between 0 and 2 indicates no obvious visible color change.

### 2.11. Sensory Evaluation Protocol

The sensory evaluation protocol was reviewed and approved by the Medical Ethics Committee of Jiangsu University (approval No. JSDX20260521002). The sensory analysis was performed with reference to the method described by Tahir et al. [31], with slight modifications. The evaluation panel consisted of ten graduate students (five males and five females, aged 20–30 years), all of whom were familiar with the principles of sensory evaluation and had completed initial training on the evaluation criteria and sample handling procedures. Sugar-impregnated hawthorn samples from different treatment groups were coded with random three-digit numbers and served on pure white plates at room temperature (25 ± 2 °C). Evaluators assessed the samples under natural daylight. Before evaluation, panelists inspected each sample for any foreign objects. Panelists rinsed their mouths with mineral water between samples to avoid carryover effects. Each panelist evaluated the color, aroma, taste, texture, and overall acceptability of the samples according to the criteria detailed in Supplementary Table S1. All samples were evaluated in triplicate by each panelist, and the scores were averaged.

### 2.12. Statistical Analysis

All experiments were performed in at least triplicate. Results were expressed as mean ± standard deviation (SD). Statistical analyses were performed using IBM SPSS Statistics 27 (IBM Corp., Armonk, NY, USA). Data were analyzed by one-way analysis of variance (ANOVA) followed by Tukey’s post hoc test, with a significance level of *p* < 0.05. Data visualization was conducted with Origin 2021 (OriginLab Corporation, Northampton, MA, USA), and schematic diagrams were created using Inkscape (version 1.4.3, Inkscape Community). Porosity was calculated using ImageJ (version 1.54r, National Institutes of Health, USA). Photographic images were captured using a digital camera (E8, aigo, Beijing, China). Image processing, including cropping, resolution adjustment, and compression, was performed using PhotoScape X (version 4.2.1, Mooii Tech, Cheonan-si, Chungcheongnam-do, Republic of Korea).

## 3. Results and Discussion

### 3.1. Selection of Ultrasonic Power for Cell Structure Modification

**Figure 2:**
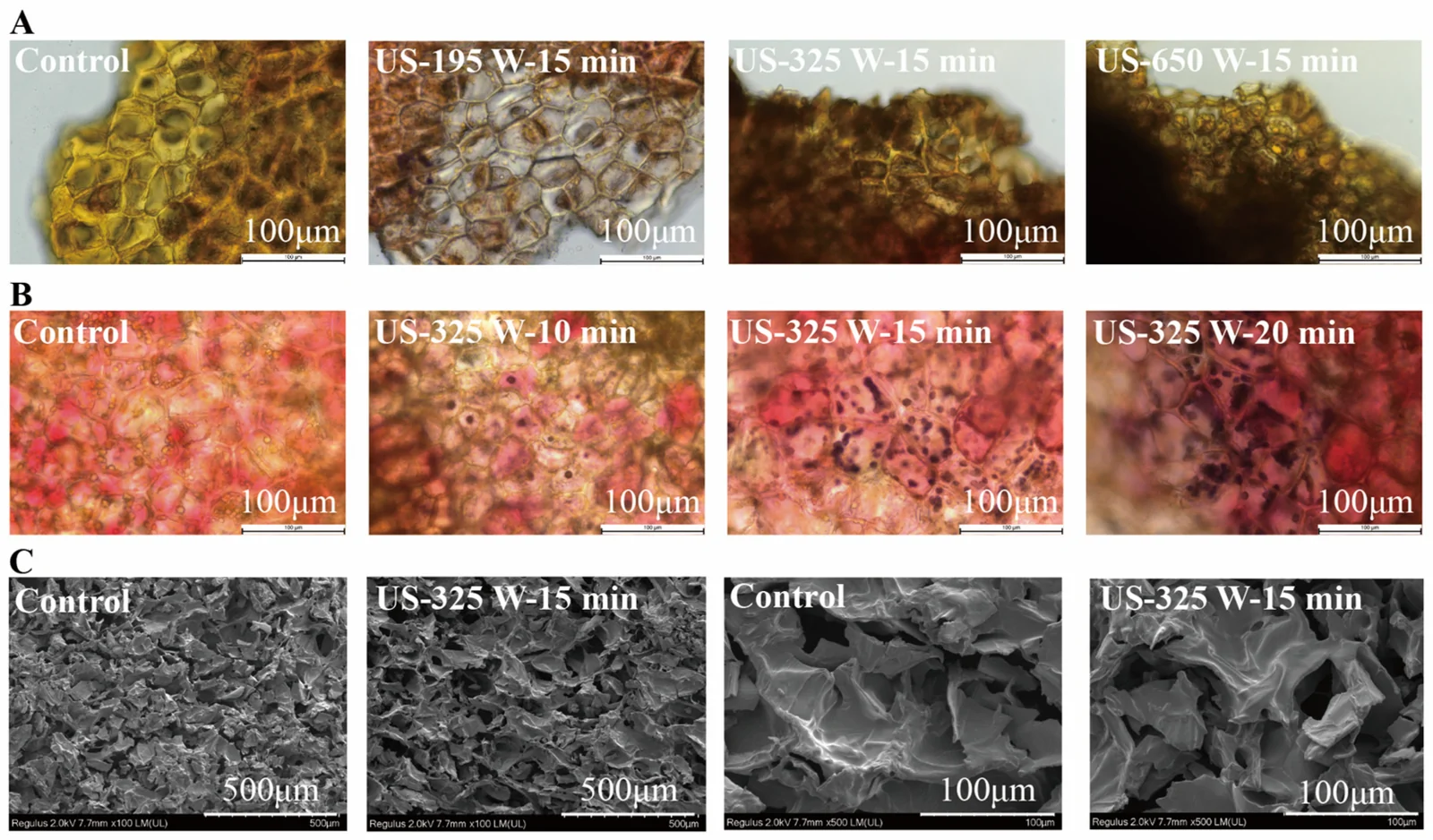
Microstructural changes before and after the ultrasonic treatment of hawthorn. (**A**) Microscopic images of epidermal cells, (**B**) Mesocarp cells stained using iodine solution (starch granules) (The red coloration in the images is attributed to the natural pigments present in hawthorn tissue), and (**C**) SEM images of the porous structures at two magnifications—100× and 500×.

The microscopic images of hawthorn surface cells are presented in Figure 2A. The ultrasonic cell disruption instrument was operated at a frequency of 20–25 kHz with a processing time of 15 min. In the untreated control (0 W), the tissue morphology was preserved, showing prominent cell boundaries and well-defined cell organization. Following US treatment at 195 W, the cell arrangement was quite compact, and no noteworthy changes were seen in the structure when compared to the control. Then, when the ultrasound power was increased to 325 W, the cell boundaries became markedly blurred, indicating cellular damage and enlarged intercellular spaces. At 650 W, although the tissue architecture appeared relatively intact at the macroscopic level, this was primarily due to the blockage of intercellular spaces caused by excessive cell wall fragmentation and the leakage of viscous intracellular substances, which masked the underlying cellular damage. Overall, these results suggest that ultrasound can disrupt cell structures and increase intercellular spaces under certain power conditions, indicating its potential effectiveness in this context [32]. However, excessive US power had a counterproductive effect, leading to cell fragmentation and the leakage of viscous substances such as pectin polysaccharides, which filled and blocked the intercellular spaces and pores [33]. Based on these observations, 325 W was selected as the appropriate US power under the conditions tested.

The three-dimensional (3D) cell imaging of hawthorn flesh cells is shown in Figure 2B, where the internal particles are starch granules [34]. In the untreated control group (0 min), the cell membranes remained intact and the cells maintained their structure with a tight arrangement. After 10 min of US treatment, a few starch granules were stained by iodine, and the cell boundaries began to lose their definition. When the treatment time was extended to 15 min, more starch granules were stained, and significant cell damage was observed. After 20 min of treatment, a large amount of starch was stained dark. The cell membranes were severely damaged, but the cell outlines remained visible, and the morphology was well preserved with a compact arrangement. These results indicate that US treatment disrupted cell membrane integrity, allowing the iodine solution to penetrate and stain the starch granules. Meanwhile, US treatment induced structural changes within a suitable time frame. However, excessively prolonged US treatment was less effective. This may be attributed to degradation of the tissue microstructure caused by extended ultrasonic exposure, leading to the detachment of cell wall fragments and the leakage of considerable amounts of intracellular pectin. These changes may result in blurred tissue structure boundaries. This finding is consistent with the conclusion of Piasecka et al. [35]. Overall, the most pronounced cellular changes occurred at a treatment time of 15 min, suggesting that this duration was the most effective among the tested durations for inducing controlled cellular disruption and enhancing mass transfer.

The SEM microscopic porous structures of freeze-dried hawthorn before and after US treatment at 325 W for 15 min are presented in Figure 2C. At a magnification of 100×, the untreated control hawthorn had a compact appearance and was characterized by a reduced number of pores and high levels of debris. The pores were narrow and locally scattered with a porosity of 16.8 ± 2.0% (based on ImageJ calculation). Irregular pores were generated after the US treatment, and both the number of pores and the porosity were increased, reaching a porosity of 19.3 ± 1.8%. With the magnification of 500×, the hawthorn without US treatment showed that the pores were stacked with cell debris, which increased markedly after US treatment. In particular, the average pore size (calculated from the captured images) increased from 30.8 ± 13.2 μm to 43.5 ± 24.5 μm. These SEM images provide further evidence of the conclusions reached in the above-described microscopic analyses that US both generated energy on the hawthorn surface by cavitation and had the ability to disrupt the surface structure.

Although ultrasonic powers of 455 W and 520 W and durations of 5 min and 25 min were also tested during preliminary screening, these conditions either yielded intermediate responses similar to those already described (455 W and 520 W) or produced changes that were either negligible (5 min) or comparable to the reported results without offering additional insights (25 min). Therefore, they were excluded from the final presentation to maintain clarity and focus on the representative findings.

### 3.2. Total Sugar Level and the Sugar Concentration Gradient Across the Cross Section

**Figure 3:**
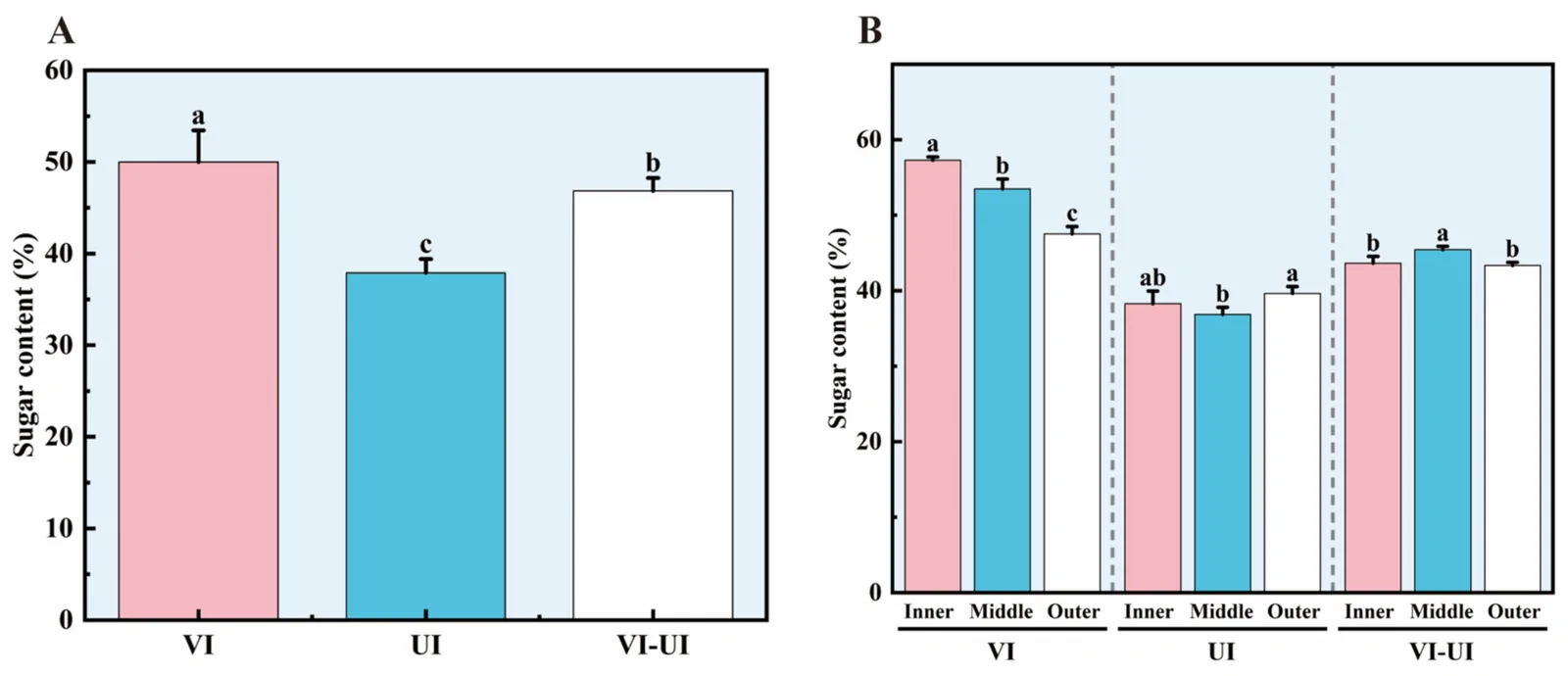
Effects of different treatments on sugar content and distribution in hawthorn samples. (**A**) Total sugar content; (**B**) cross-sectional sugar concentration gradient. Different letters indicate significant differences (*p* < 0.05).

The total sugar content and cross-sectional sugar concentration gradient of hawthorn samples under different treatment conditions are presented in Figure 3. The hawthorn treated with VI had the highest content of total sugar (about 50%) and exhibited a significant concentration gradient from the inner to middle to outer regions (*p* < 0.05). In contrast, there was no significant difference in sugar distribution among the inner, middle, and outer regions of the UI-treated hawthorn (*p* > 0.05), but the total sugar content of the UI hawthorn was the lowest (~38%). After VI-UI treatment, the total sugar level was about 47% and, likewise, no significant difference in sugar distribution among the three regions was found (*p* > 0.05).

**Figure 4:**
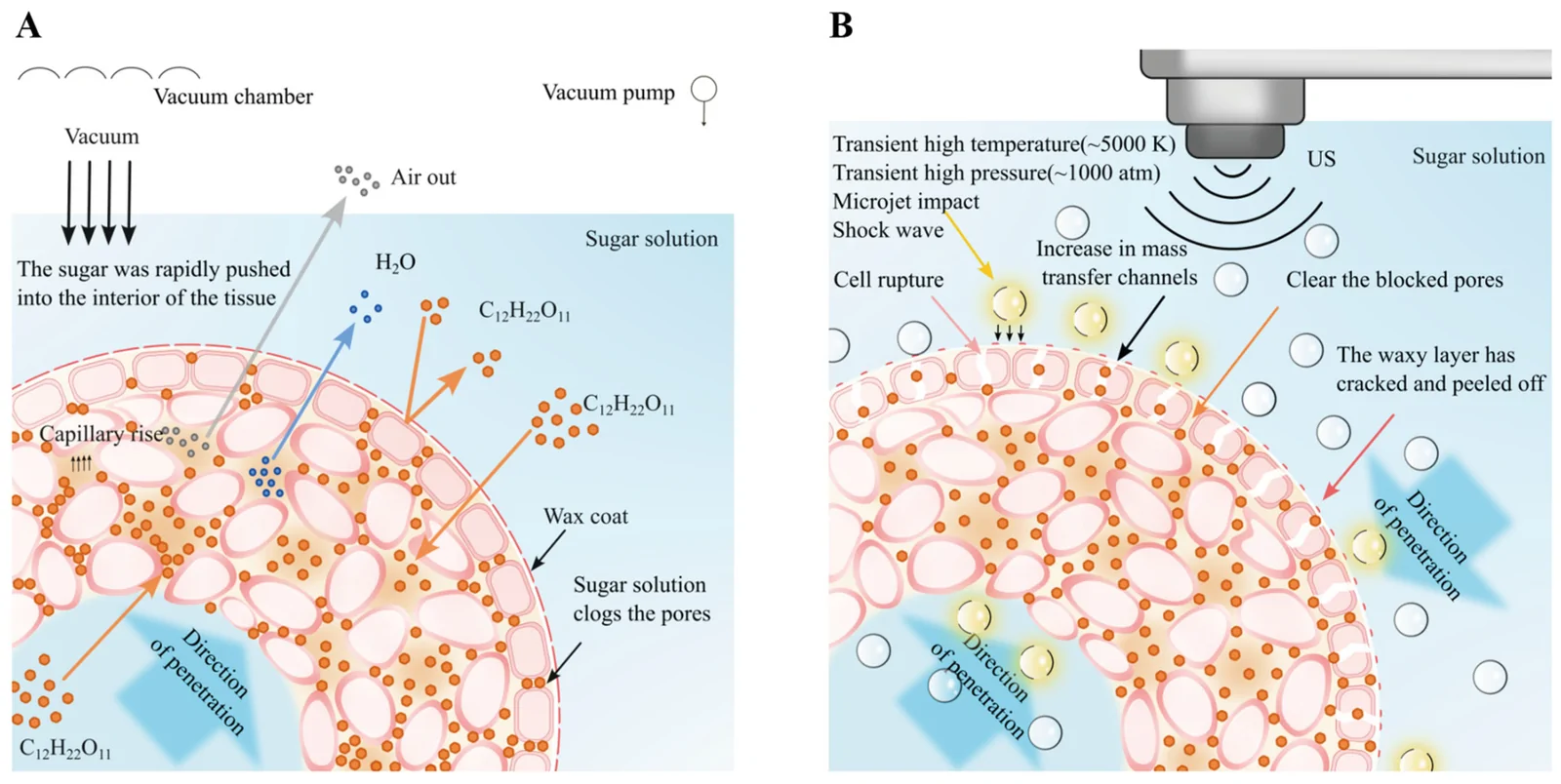
Schematic diagrams of the proposed mechanisms for different treatments. (**A**) VI treatment; (**B**) UI treatment.

Hawthorn is encased in a thick waxy coating, consisting mainly of alkanes, alcohols and long-chain fatty acids, that forms a barrier to solution permeation [36]. In this study, freeze-dried hawthorn was used as the raw material. During freeze-drying, ice crystals sublimated, forming a highly porous network structure. However, the waxy layer exhibited only minor fractures and was not completely disrupted. Meanwhile, the epidermal cell structure remained relatively intact and continued to exert a blocking effect [37]. During VI, air was expelled from the porous structure during the depressurization stage. Upon pressure restoration, the sugar solution was driven by the pressure difference and capillary forces to penetrate the tissue [38]. However, due to the difficulty of effective penetration from the exterior, the sugar content in the outer layer remained relatively low [39]. The internal structure of hawthorn is inherently dense. After freeze-drying, fine pores are formed within the tissue. Sugar accumulates and becomes trapped in the central region, which contains dense pores and exhibits a strong capillary retention effect (Figure 4A). Therefore, more sugar accumulated in the inner region, resulting in uneven distribution [40].

In contrast, sugar distribution in the UI-treated samples was more uniform across the fruit cross-section. During UI, the ultrasonic probe converts electrical energy into mechanical energy through high-frequency vibration, which is then transmitted into the sugar solution as sound waves. The resulting cavitation effects lead to the formation and instantaneous collapse of microbubbles, generating localized high temperatures, high pressures, and microjets [41]. These physical forces disrupt the epidermal cells and waxy layer of the freeze-dried hawthorn, increasing mass transfer channels and promoting sugar solution penetration from the exterior to the interior (Figure 4B). Consequently, the uniformity of sugar impregnation is improved [42,43]. However, due to the absence of a pressure difference, the overall sugar impregnation efficiency remains lower than that of VI.

### 3.3. Establishment of the Combined VI-UI Treatment Method

**Figure 5:**
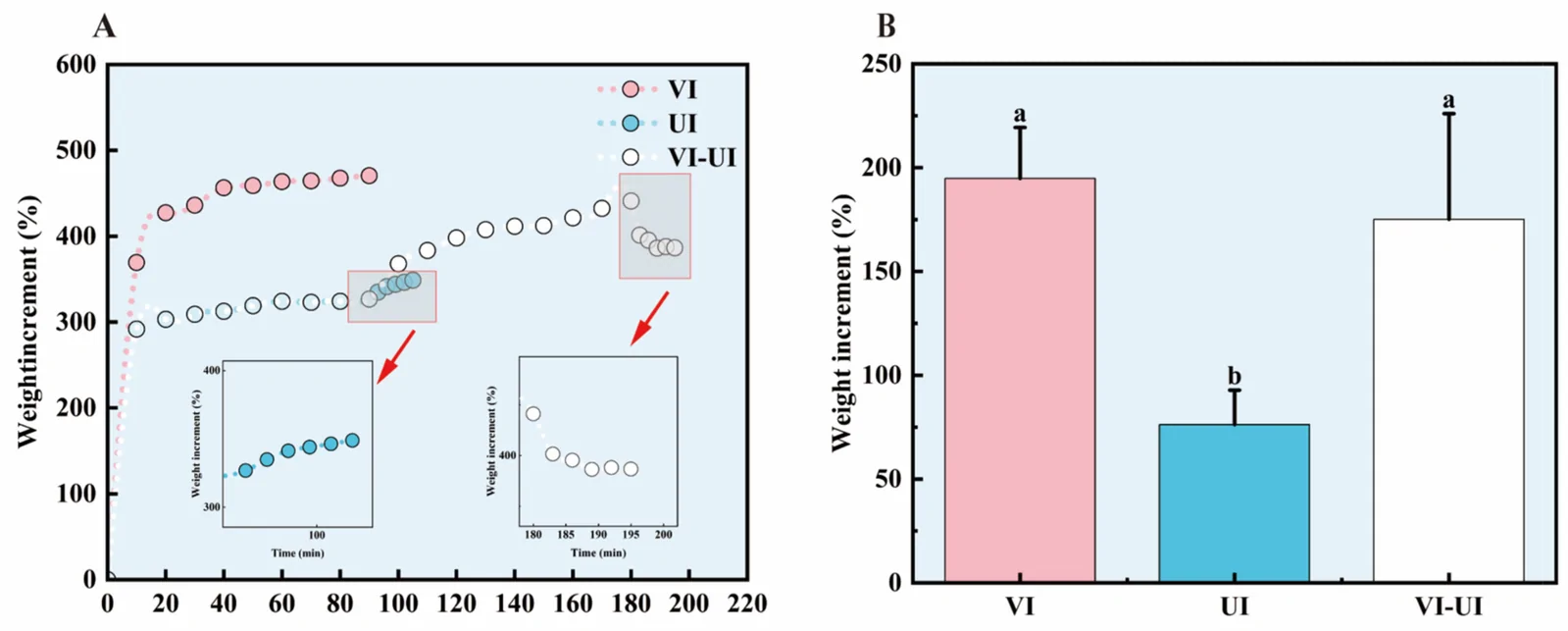
Effects of different treatments on sugar impregnation and weight gain of hawthorn samples. (**A**) Sugar impregnation rate; (**B**) weight gain. Different letters indicate significant differences (*p* < 0.05).

As shown in Figure 3 and Figure 5, each individual treatment has inherent advantages and limitations. VI treatment exhibits a significant advantage in sugar impregnation efficiency but results in poor impregnation uniformity (inner > middle > outer gradient). In contrast, UI treatment shows lower efficiency than VI but ensures better impregnation uniformity across the hawthorn cross-section. If freeze-dried hawthorn is first treated with UI, the cavitation effect disrupts the waxy layer and epidermal cells on the surface. This increases mass transfer channels. Then, when VI is applied for sugar impregnation, a large amount of sugar is forced into the pores under the pressure difference, leading to pore blockage. It also adversely affects impregnation uniformity. Even though the pore number increases, VI still plays a dominant role in uneven sugar penetration. It also plays a role in the formation of sugar gradients.

Based on these considerations, the final process flow was established as follows (illustrated in the graphical abstract). The pre-soaking step was separated from the UI step. Then the VI step was inserted between them. The pre-soaking step serves as a controlled auxiliary method that not only creates favorable mass transfer conditions for subsequent ultrasonic impregnation but also limits excessive sugar uptake by preventing the sugar solution from being forced too rapidly into the tissue during the subsequent VI step. This allows the high-concentration sugar solution to completely impregnate the freeze-dried hawthorn without overloading the internal structure. Subsequently, VI treatment rapidly delivers a large amount of sugar solution into the tissue. Finally, UI treatment is applied to clear the pores that were blocked by the sugar solution during the VI process, resulting in even distribution of internal sugar [44,45]. Following the combined VI-UI treatment, the impregnation rate was at a medium level (Figure 5A), which is consistent with the weight gain results in Figure 5B. Meanwhile, the sugar distribution inside the samples was uniform (Figure 3B), confirming the effectiveness of the established sequential treatment protocol.

### 3.4. Changes in Cellular Microstructure

**Figure 6:**
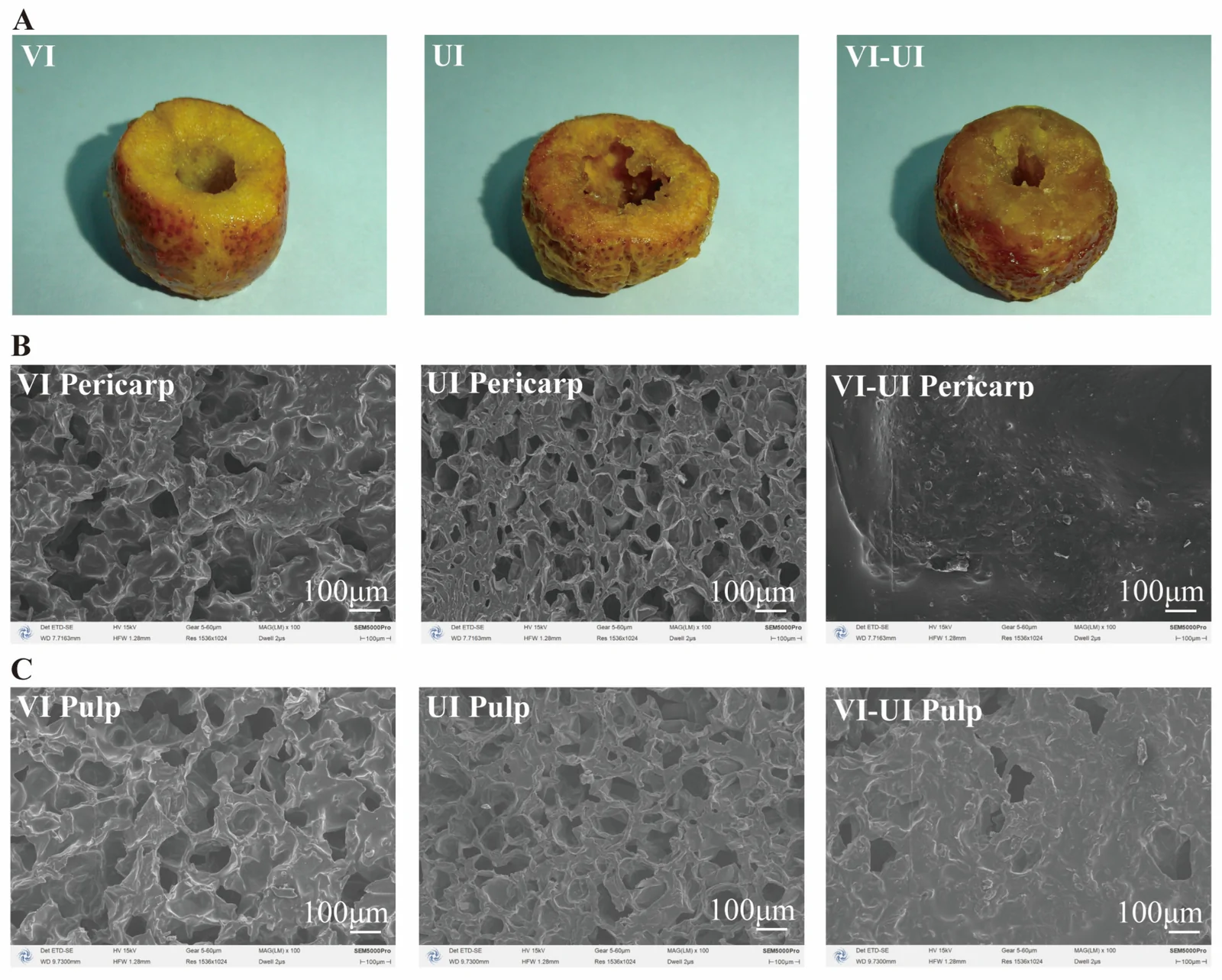
Morphological and microstructural characteristics of hawthorn samples under different treatments. (**A**) Photographs of samples from different treatment groups; (**B**) SEM images of the pericarp; (**C**) SEM images of the pulp.

The direct view of the hawthorn samples is presented in Figure 6A. The VI-treated samples appeared overall yellowish, with a plump and firm texture, and exhibited the highest sugar uptake. In contrast, the UI-treated samples showed obvious surface wrinkling and the lowest sugar uptake. The VI-UI-treated samples displayed a bright, plump color, appeared softer and more pliable, and demonstrated better sugar uptake compared to the UI-treated samples.

The SEM micrographs of the hawthorn samples are presented in Figure 6B (pericarp) and Figure 6C (pulp). The pericarp of the VI group remained relatively intact with no obvious rupture. It exhibited a pore diameter of 126.7 ± 38.4 μm (calculated from the captured images). It also had a porosity of 14.6 ± 1.5% (calculated using ImageJ). In the UI group, the pore number increased. The average pore diameter was 73.8 ± 25.8 μm, and the porosity reached 26.9 ± 2.4%. The pericarp surface of the VI-UI group was smooth, presenting a continuous, film-like dense structure with no observable pores. This is because during VI treatment, the sugar solution gradually and directionally penetrated from the flesh to the skin. The sugar was mainly distributed within the pores, which resulted in minimal changes to the pericarp structure. During UI treatment, the cavitation effect caused bubble collapse. This generated localized high pressure and microjets. These forces strongly impacted the cell walls and membranes. This led to cell structure fragmentation and the formation of larger, irregular pores. At the same time, ultrasonic vibration promoted sugar diffusion, and the generated shear forces further disrupted intercellular connections. This enhanced pore connectivity, caused some pore walls to fracture, and led to pore fusion and expansion [46]. During VI-UI treatment, the hawthorn skin was subjected to sugar solution penetration from the flesh to the skin during the VI stage. It was also subjected to bidirectional penetration (skin to flesh and flesh to skin) during the UI stage. This caused swollen cells to deform under the combined effects of cavitation and internal pressure. This led to cell wall adhesion and mutual squeezing. In the end, they formed a dense membranous structure [47].

Regarding the fruit pulp, the pores were clear and uniform in the VI-treated samples. They had a smooth surface, a continuous structure, and a loose porous network. The average pore diameter was 132.3 ± 68.8 μm, and the porosity was 18.7 ± 3.5%. This indicates that penetration of the high-concentration sugar solution effectively supported the porous structure. In the UI-treated samples, the pore number increased, but the shapes were irregular. The average pore diameter was 111.8 ± 32.0 μm, and the porosity reached 19.1 ± 1.5%. These features were primarily attributed to cavitation-induced tissue damage. Compared with the pericarp, the pulp exhibited lower pore content, likely because cavitation energy predominantly acted on the pericarp surface. In the VI-UI-treated samples, the pore content decreased significantly to 6.6 ± 2.3%, while the average pore diameter was 129.7 ± 41.8 μm. Similar to the pericarp structure, this resulted from the combined effects of ultrasound vibration, shear forces, and cell swelling and compression induced by the sequential VI-UI treatment. Despite the marked reduction in overall pore content, the relatively large average pore diameter (129.7 ± 41.8 μm) facilitated the uniform distribution and efficient mass transfer of the sugar solution, thereby contributing to the superior overall quality of the VI-UI treated samples.

### 3.5. Water Distribution and Water Activity

**Figure 7:**
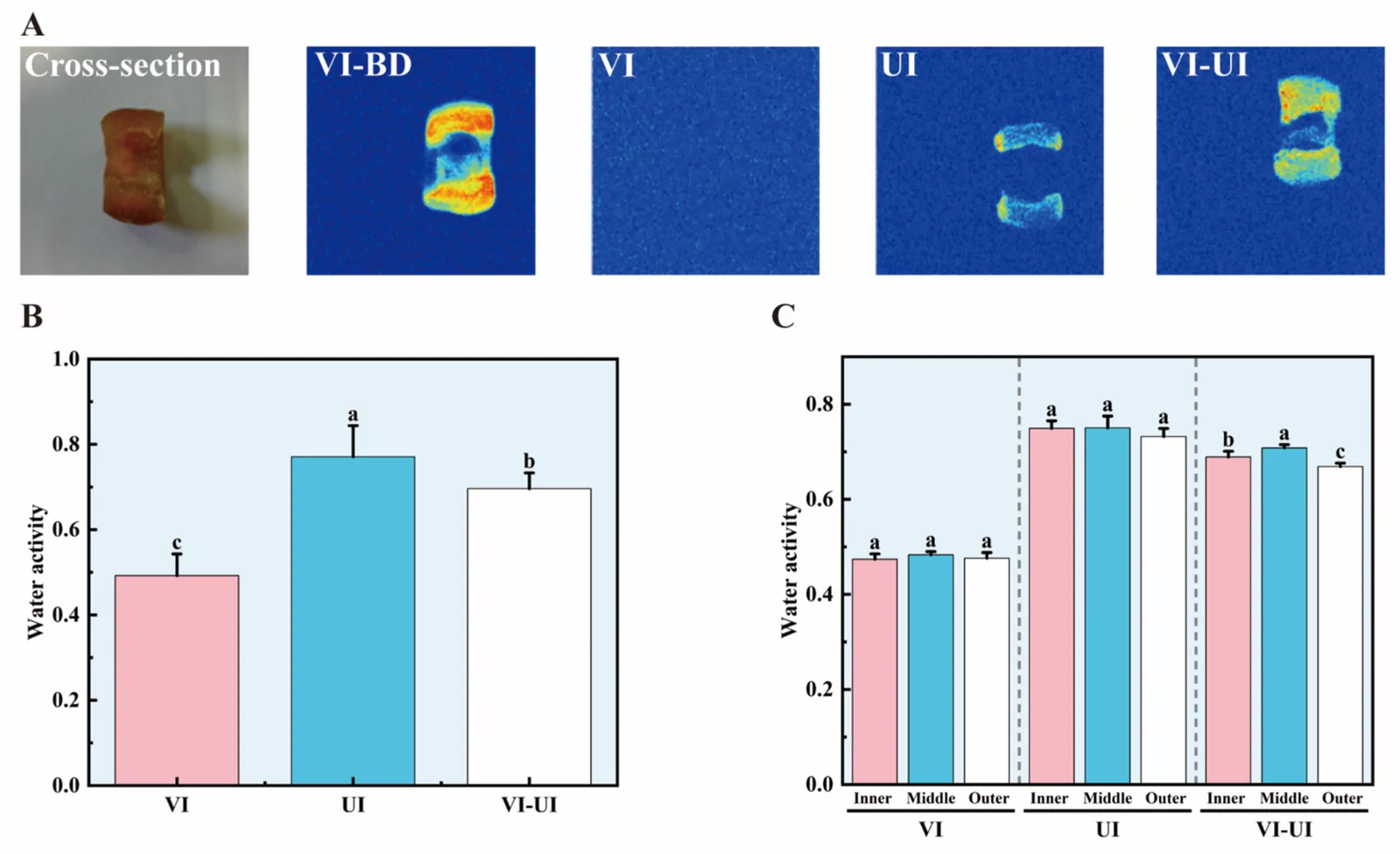
Water distribution and water activity of hawthorn samples under different treatments. (**A**) Moisture content distribution. The pseudo-color scale indicates the NMR signal intensity, where red represents high signal and blue represents low signal; (**B**) overall water activity; (**C**) cross-sectional water activity gradient. Different letters indicate significant differences (*p* < 0.05).

The water distribution of hawthorn samples measured by low-field NMR imaging is presented in Figure 7A, where warmer colors indicate higher water content. Since the VI group exhibited low water activity, its NMR signal was too weak to be detectable (a_w_ = 0.49); therefore, VI samples before drying (VI-BD) were included for comparison.

In the VI-BD samples, water distribution was higher in the middle region and lower on both sides (Figure 7A). During sugar impregnation, the pressure difference drove the sugar solution into the tissue, preferentially filling low-resistance areas and displacing moisture. The middle region exhibited higher water activity after processing, as moisture loss from this central area was more difficult during the subsequent vacuum drying step. The internal region had dense pores and strong capillary retention. Therefore, it retained more sugar solution. This resulted in lower water activity. In contrast, the external region had high mass transfer resistance. Therefore, it retained more moisture. After vacuum drying, moisture loss occurred mainly in the internal and external regions. This left slightly higher moisture levels in the middle region.

The water distribution in the middle and internal regions of the hawthorn treated with UI was uniform. The only exception was in the external region where moisture content was lower. Microjets were created by ultrasound cavitation. These disturbed the waxy layer and made the epidermal cells weaker. This increased mass transfer channels and decreased the resistance in the system. Therefore, it slowed down the formation of sugar gradient. The sugar solution was plentiful in both the internal and external areas. It bound water in a stable hydration structure with close water contact [48]. The free water reduced after vacuum drying. This led to the reduction in water activity. However, there was not much deep penetration with the UI treatment. This resulted in a decreased sugar level in the center. Therefore, more moisture was present there.

The VI-UI-treated hawthorn samples had the most uniform water distribution. Only the middle region had a slightly higher water content. This uniformity resulted from the combined effects of vacuum and ultrasound treatment. VI treatment first created a sugar gradient of “inner > middle > outer” (Figure 3B). Subsequently, cavitation generated by UI treatment weakened the surface barrier, accelerated mass transfer, and increased external sugar impregnation, thereby preventing the formation of pronounced gradient. During vacuum drying, moisture in the middle region was less easily lost, resulting in slightly higher water activity there. However, the overall difference across the cross-section was minimal. Compared with VI or UI treatment alone, the uniformity of water distribution was significantly improved in the VI-UI-treated hawthorns [49].

The water activity of hawthorn samples is presented in Figure 7B,C. Overall, water activity distribution was uniform in the VI- and UI-treated hawthorn samples. Among all treatment groups, the UI-treated samples showed the highest water activity (approximately 0.77 a_w_). The VI-UI-treated hawthorn displayed intermediate water activity with uneven distribution pattern: higher in the middle region and lower on both sides. Consistent with the sugar concentration data presented in Figure 3, water activity was negatively correlated with sugar concentration. This is because sugar molecules bind to water molecules via hydrogen bonds, converting free water into bound water and hence reducing water activity [50]. Different treatments exhibited distinct effects on the water state (Supplementary Figure S2).

### 3.6. Mechanisms Underlying Overall Uniformity Differences

Following the analysis of cross-sectional sugar and water distribution, the impact of different treatments on overall uniformity (i.e., consistency among different individual samples) was investigated. Given the significant natural variation among hawthorn fruits, minimizing inter-individual variability is particularly important for product quality control [51]. The overall uniformity scores for total sugar content, hardness, moisture content, pectin, and color difference are summarized in Table 1. The VI-treated hawthorn exhibited an OUI of 50.99 (medium); the UI-treated hawthorn achieved an OUI of 60.69 (good); and the VI-UI group achieved an OUI of 79.65 (excellent), representing an improvement compared with VI or UI treatment alone.

During VI treatment, the pressure difference is controllable and the mass transfer direction is consistent. Although sugar leakage shows little variation among samples, individual differences in hawthorn (e.g., porosity, pore size, and tissue density) still affect impregnation efficiency [52]. VI has a weak effect on cell wall damage, and pectin loss occurs primarily via concentration gradient diffusion. Consequently, residual pectin content depends largely on the raw material, leading to substantial inter-individual differences. Due to variations in pore size and connectivity, sugar leakage during VI treatment is highly dependent on pore structure. In samples with good pore connectivity, the sugar solution fully fills the pores, providing enhanced support; in samples with poor connectivity, filling is insufficient, resulting in weaker support. Consequently, the total sugar content showed relatively lower overall uniformity than the other two groups, and hardness exhibited relatively greater variation among samples [53]. Brightness and color are affected by sugar impregnation efficiency: the more fully the sugar infiltrates, the higher the brightness, and the greater the individual differences. Therefore, the VI group has the lowest OUI, indicating the worst uniformity.

UI treatment primarily relies on cavitation to enhance mass transfer, lacking a pressure difference as a driving force. Individuals with loose tissue undergo rapid sugar impregnation and dehydration, while those with dense tissue show the opposite trend. This results in greater inter-individual differences in water content and hardness, leading to poor overall uniformity [54].

In the VI-UI treatment, VI first uses pressure difference to inject a high-concentration sugar solution, completing bidirectional exchange of sugar and water; subsequently, UI clears blocked pores to evenly distribute sugar and water within the tissue. This combined approach avoids the limitations of VI (being affected by pore structure differences) and overcomes the uneven dehydration characteristics of UI, resulting in smaller inter-individual differences in total sugar content, hardness, water content, and color difference. Ultimately, the VI-UI treatment achieved the best overall uniformity.

### 3.7. Sensory Evaluation Results

**Figure 8:**
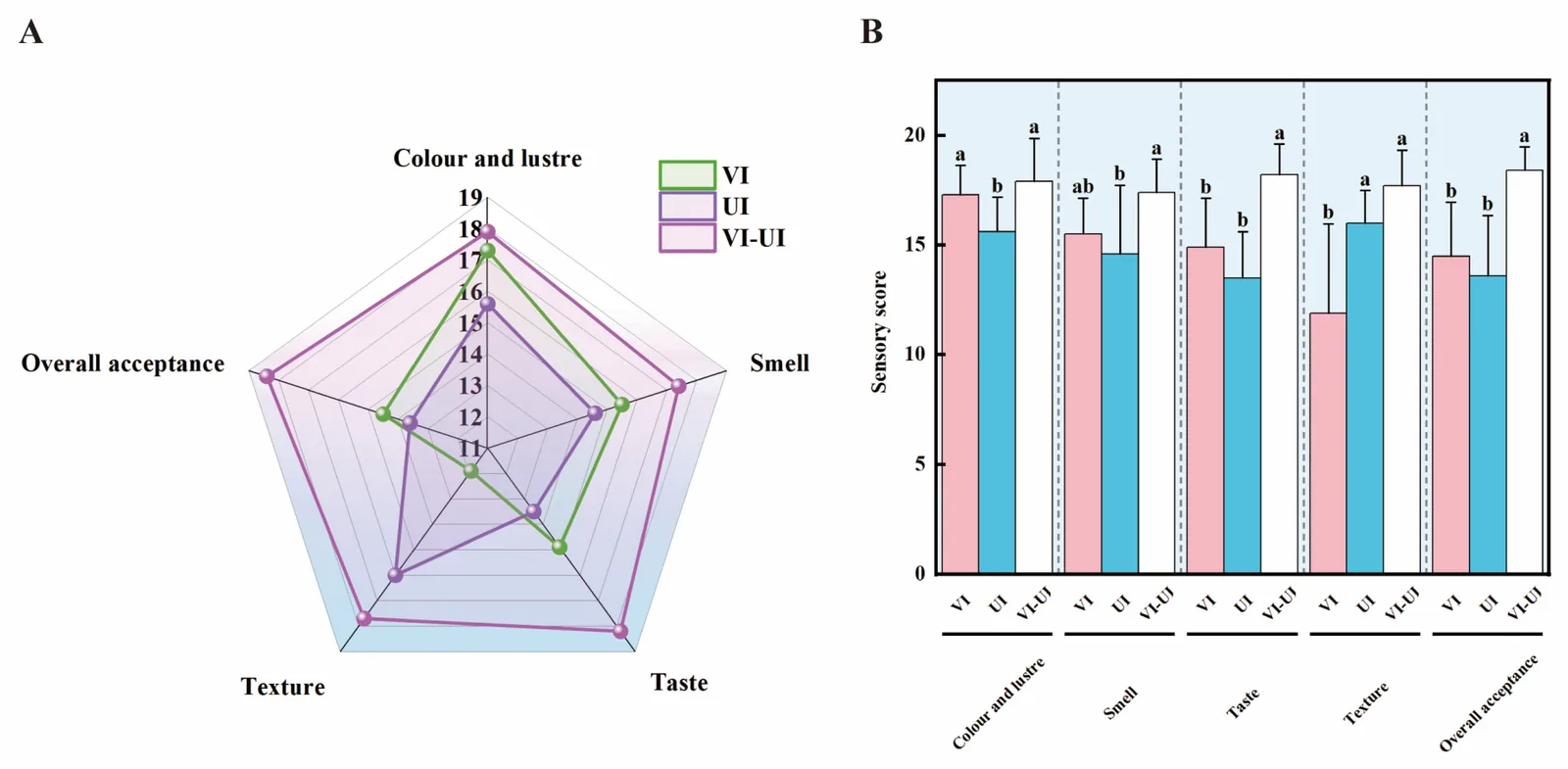
Sensory evaluation of hawthorn samples under different treatments. (**A**) Radar chart; (**B**) Overall acceptance scores. Different letters indicate significant differences (*p* < 0.05).

The sensory evaluation results (*n* = 10 panelists) revealed that the VI-UI group received the highest taste scores (18.2). The hawthorn samples from this group exhibited a harmonious blend of sweet and sour flavors and were highly palatable, further demonstrating the uniformity of sugar infusion achieved by the combined treatment. The combined application of VI and UI not only ensured an appropriate amount of sugar uptake but also guaranteed uniformity both within individual samples (cross-sectional consistency) and among different samples (inter-individual consistency). Compared with hawthorn samples treated with VI or UI alone, the VI-UI treated samples showed marked improvements in both taste and texture. The taste scores increased from 14.9 (VI group) and 14.5 (UI group) to 18.2 (VI-UI group), and the texture scores increased from 11.9 (VI group) and 16.0 (UI group) to 17.7 (VI-UI group) and the overall acceptance rate increased from 14.5 (VI group) and 13.6 (UI group) to 18.4 (VI-UI group), indicating a significantly higher overall acceptance for the VI-UI treatment (*p* < 0.05, Figure 8).

## 4. Conclusions

VI primarily relies on pressure-driven capillary action, which confers high sugar impregnation efficiency (50% total sugar) but inevitably creates a distinct “inner > middle > outer” sugar concentration gradient. In contrast, UI disrupts the surface using cavitation to enable uniform penetration, but at the cost of lower overall efficiency (38% total sugar). The combined VI-UI treatment complements the benefits of both treatments: VI provides rapid sugar delivery into the deep tissue, while UI removes pore blockages and improves distribution uniformity. This combined approach achieved a moderate total sugar content of 47% with uniform distribution, and significantly enhanced surface porosity (from 16.8% to 19.3%) and average pore diameter (from 30.8 μm to 43.5 μm) after ultrasonic pretreatment. SEM analysis further revealed that VI-UI treatment transformed the skin into a dense, non-porous structure while reducing pulp porosity from 18.7% (VI) and 19.1% (UI) to 6.6%, with the average pore diameter remaining relatively large (129.7 μm), which facilitated uniform sugar distribution and efficient mass transfer. The quantitative data validate the VI-UI treatment as the one with the best overall uniformity (OUI = 79.65), which was higher than VI (50.99) and UI (60.69) alone, and also resulted in the best sensory acceptance, with the highest scores for taste (18.2), texture (17.7), and overall acceptance (18.4). Overall, VI-UI is an effective processing method for obtaining high-quality fruit products with uniform impregnation.

## Figures and Tables

**Table 1:** Overall uniformity scores of hawthorn samples.

Samples	Tests	*CV* (%)	Score (0–100)	Proportion (%)	OUI
VI	Total sugar content	5.47	96.85	30	50.99
	Hardness	17.01	19.91	20	
	Water content	15.43	30.45	25	
	Pectin content	9.67	68.90	15	
	Chromatic aberration	24.31	0	10	
UI	Total sugar content	3.97	100	30	60.69
	Hardness	28.24	0	20	
	Water content	13.46	43.60	25	
	Pectin content	1.51	100	15	
	Chromatic aberration	12.81	47.95	10	
VI-UI	Total sugar content	3.06	100	30	79.65
	Hardness	16.65	22.33	20	
	Water content	5.42	97.17	25	
	Pectin content	8.90	74.03	15	
	Chromatic aberration	5.32	97.88	10	

## Data Availability

The original contributions presented in this study are included in the article/Supplementary Material. Further inquiries can be directed to the corresponding author.

## Supplementary Materials

The following supporting information can be downloaded at: https://www.mdpi.com/article/10.3390/foods15162873/s1, Figure S1: Standard curve of pectin expressed as galacturonic acid equivalent.; Figure S2: Effects of different treatments on the water state; Table S1: Sensory evaluation scoring criteria for hawthorn samples.
