# Supplementary material for: Surface Modification and Sugar Infusion of Freeze-Dried Hawthorn via Combined Vacuum-Ultrasound Treatment
Source: Foods. 2026 Aug 17;15(16):2873. doi: 10.3390/foods15162873 (PMC13512562; doi:10.3390/foods15162873)
Supplement: Supplementary file 1 [file foods-15-02873-s001.zip › Figure S1.pdf]

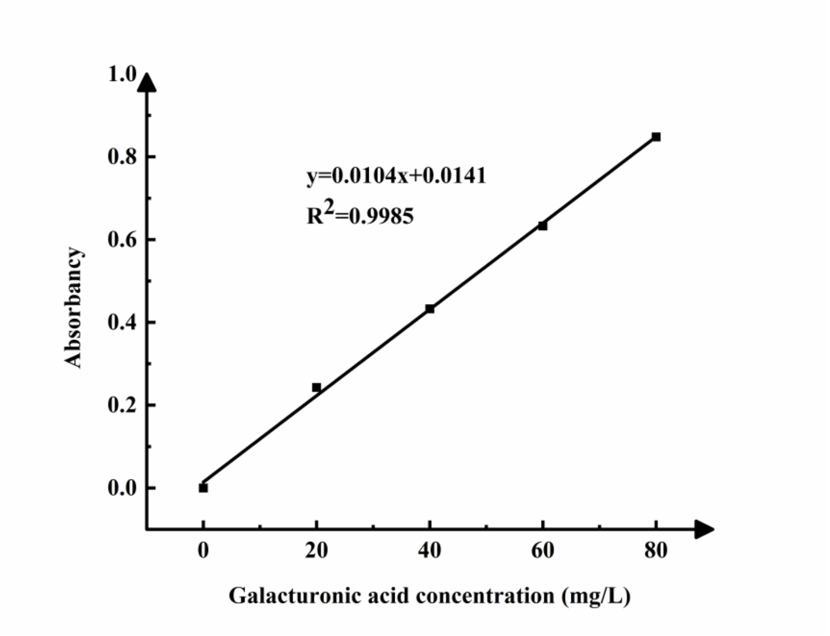

Figure S1. Standard curve of pectin expressed as galacturonic acid equivalent.
