# Supplementary material for: Surface Modification and Sugar Infusion of Freeze-Dried Hawthorn via Combined Vacuum-Ultrasound Treatment
Source: Foods. 2026 Aug 17;15(16):2873. doi: 10.3390/foods15162873 (PMC13512562; doi:10.3390/foods15162873)
Supplement: Supplementary file 1 [file foods-15-02873-s001.zip › Table S1.pdf]

Table S1. Sensory evaluation scoring criteria for hawthorn samples.

Attribute (score)	Grade	Score range	VI	UI	VI-UI
Colour and lustre （20）	Poor: dark, dull, spots or browning	0-7			
	Acceptable: basically normal, no obvious defects	8-14			
	Excellent: pure, uniform, appetizing	15-20			
Smell （20）	Abnormal: burnt, fermented or musty; no fruity aroma	0-7			
	Plain: no off-odors, but fruity notes not prominent	8-14			
	Rich: pure fruit fragrance, strong, no unpleasant odor	15-20			
Taste （20）	Imbalanced: too sour or too sweet, abrupt	0-7			
	Basically balanced: well-integrated, slightly more sour or sweet	8-14			
	Balanced: perfectly blended, pleasant, long aftertaste	15-20			
Texture （20）	Poor: too tough, hard to chew	0-7			
	Acceptable: moderately firm/soft, easy to chew, slightly tight	8-14			
	Good: moderately firm/soft, tender, juicy, fine and compact	15-20			
Overall acceptance （20）	Poor: unacceptable, strongly disapproved	0-7			
	Generally: accepted reluctantly, neither beneficial nor harmful	8-14			
	Better: highly recommended, highly praised	15-20			
